# Bowel Ischemia Secondary to Campylobacter Enterocolitis: A Case Series and Review of the Literature

**DOI:** 10.7759/cureus.39183

**Published:** 2023-05-18

**Authors:** Ryan Teh, Daniel Lee, Yeow Chun Tee, Tulsi Menon

**Affiliations:** 1 General Surgery, Fiona Stanley Hospital, Perth, AUS

**Keywords:** infectious colitis, portal venous gas, pneumatosis intestinalis, bowel ischemia, campylobacter enteritis

## Abstract

Campylobacter is the most common cause of diarrheal illness worldwide and is generally self-limiting. We present two cases of Campylobacter enterocolitis complicated by bowel ischemia in a 79-year-old male and a 53-year-old male, both presenting with abdominal pain and diarrhea and elevated lactate and C-reactive protein (CRP) levels. CT demonstrated common findings of pneumatosis intestinalis (PI) and portal venous gas. Exploratory laparotomy performed on the former demonstrated extensive small bowel infarction that was noncompatible with life and he was palliated postoperatively. The latter improved clinically after resection of the ischemic section of the small bowel with primary stapled anastomosis and closure. Clinicians need to be aware of the potentially fatal complications of Campylobacter-associated enterocolitis and should maintain a high clinical index of suspicion so that early surgical intervention can be considered in this patient population.

## Introduction

Campylobacter species is now recognized as the predominant microbial cause of diarrheal illness worldwide based on an early study by Skirrow in 1977 [1,2]. Campylobacter enterocolitis has a reported incidence of approximately 110 per 100,000 cases of foodborne infections per year [3] and is classically regarded as a self-limiting disease, with the site of infection commonly being the jejunum and ileum. Its management is primarily conservative, focusing on hydration and electrolyte replacement, with macrolide antibiotics mainly considered in high-risk patients such as the elderly or the immunocompromised [4].

Despite the benign nature of this common infection, unusual findings and potentially fatal complications have been reported in the literature. Pneumatosis coli on CT secondary to Campylobacter enteritis has previously been reported, in an unwell 47-year-old male, who was tachycardic and febrile. The patient was managed nonoperatively with antibiotics, with clinical improvement associated with the resolution of pneumatosis coli, as demonstrated on repeat CT [5].

Another case of Campylobacter colitis was reported in a 55-year-old female, with a background of Child-Pugh B liver cirrhosis secondary to hepatitis C and alcohol abuse, associated with toxic megacolon and sepsis [6]. CT demonstrated no evidence of intramural or free gas. The patient underwent an emergency subtotal colectomy with the formation of an end ileostomy and distal sigmoid mucous fistula. No perforation or ischemia was found. Despite initial clinical improvement, the patient deteriorated on postoperative day three with acidosis, respiratory failure, and liver failure, subsequently passing away on day four [6].

We describe two cases of Campylobacter enterocolitis-associated bowel ischemia confirmed during laparotomy. One patient, unfortunately, passed away, while the other required small bowel resection and a prolonged hospital stay.

## Case presentation

Patient one

A 79-year-old male was initially admitted to the Cardiology unit for the management of Wellens syndrome. His background was significant for pancreatic insufficiency, hypertension, aortic root aneurysm, a bicuspid aortic valve with aortic stenosis, and previous anterior resection for colorectal adenocarcinoma. Inpatient coronary angiogram and transthoracic echocardiogram demonstrated significant two-vessel disease and severe aortic stenosis. The patient was scheduled for a Bentall procedure and coronary artery bypass graft.

**Figure 1:**
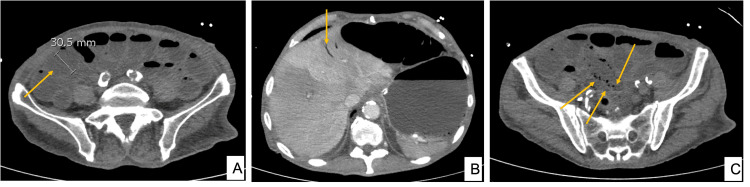
CT of patient one The images demonstrate dilated small bowel loops (A), with pneumatosis intestinalis (B), along with portal vein gas (C) CT: computed tomography

As intermittent loose stools were observed during his admission, the patient was investigated with enteric polymerase chain reaction (PCR), and he tested positive for the Campylobacter gene. This was managed with a three-day course of oral azithromycin. He subsequently deteriorated in the ward with worsening hypotension and abdominal pain. Lactate was elevated up to 8.1 mmol/L, with a white cell count of 16 x 10^9^/L and C-reactive protein (CRP) levels of 414 mg/L. The patient required metaraminol administration to maintain his mean arterial pressure. The antibiotic regimen was changed to intravenous piperacillin/tazobactam after consultation with the Infectious Diseases team. CT of the abdomen and pelvis was performed (Figure 1), demonstrating dilated bowel loops up to 31 mm with no mechanical transition point demonstrated. Small bowel pneumatosis intestinalis (PI) with portal venous gas was noted and his coeliac and superior mesenteric arteries (SMA) were patent. Overall, these features were concerning for small bowel ischemia.

Laparotomy was performed due to his ongoing deteriorating state, with worsening hypotension and increasing peritonism. Intraoperatively, approximately 40 cm of infarcted small bowel was noted, along with patchy necrosis involving the extent of the remaining small bowel. This was deemed nonsurvivable, and the decision was made to abandon further interventions. The patient was consequently placed on palliative measures and he passed away two hours postoperatively.

Patient two

A 53-year-old male presented to the emergency department with a three-day history of abdominal pain, diarrheal illness, nausea, and fever. His medical history was significant for insulin-dependent diabetes mellitus, hyperthyroidism, and hypercholesterolemia.

**Figure 2:**
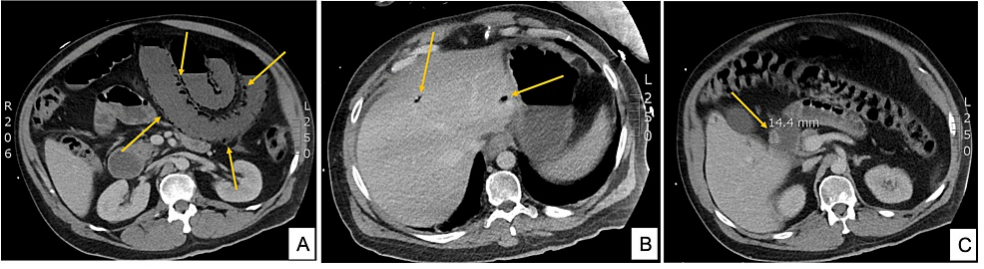
CT of patient two The images demonstrate extensive pneumatosis of the jejunum and ileum (A), portal vein gas (B), and an incidental 14 mm non-obstructing mural lesion within the first part of the duodenum (C) CT: computed tomography

A CT scan was performed given his examination findings of generalized peritonism and further blood tests showing an elevated lactate of 2.2 mmol/L, white cell count of 4.9 x 10^9^/L, and CRP of 128 mg/L. The CT scan demonstrated extensive PI of a significant portion of the jejunum and ileum with minimal small bowel enhancement, suggestive of small bowel ischemia (Figure 2). Gas was also identified within distal branches of the portal vein, as well as tributaries of the superior mesenteric vein (SMV). The coeliac artery, SMA, and inferior mesenteric arteries were patent.

**Figure 3:**
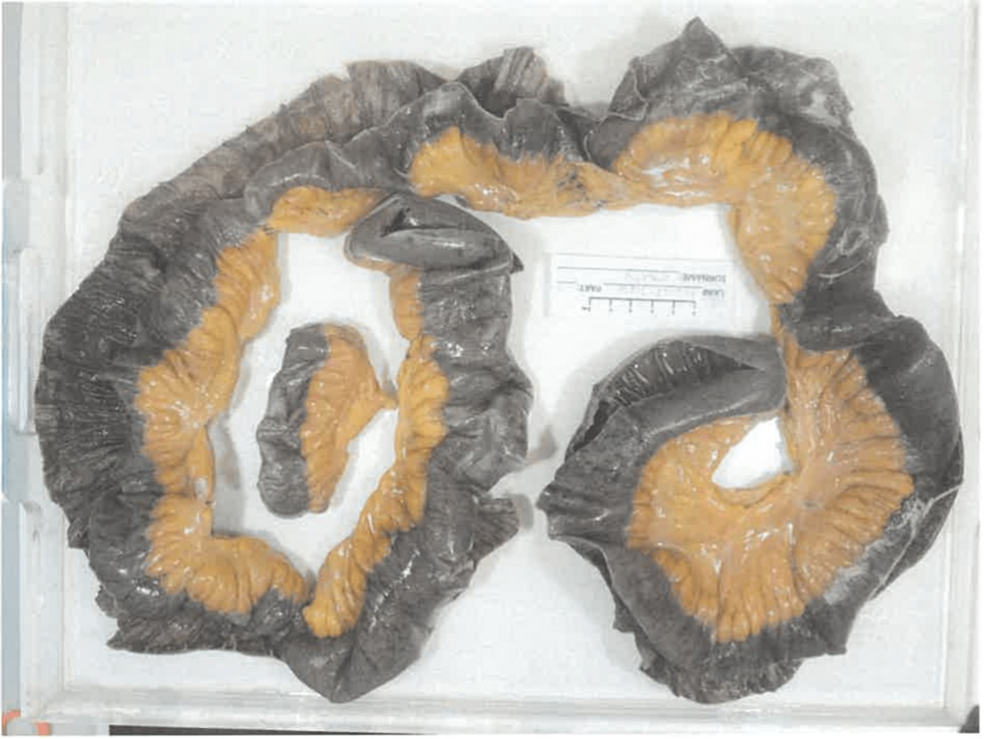
Anatomical pathology sample demonstrating necrotic small bowel in patient two

The patient underwent an emergency exploratory laparotomy where 1.7 m of ischaemic small bowel was deemed not viable and was resected (Figure 3). A strong SMA pulse was palpated intraoperatively. A laparostomy was performed and the patient was admitted to the ICU for inotropic support given his overall physiological instability. He was placed on intravenous piperacillin/tazobactam and total parenteral nutrition in the postoperative period. Stapled anastomosis of the small bowel with primary abdominal closure was performed the next day. The patient required a total of four days of ICU care for monitoring and weaning off inotropic support.

Enteric PCR returned with Campylobacter and the patient was started on a three-day course of oral azithromycin. Histopathology of the intraoperative small bowel specimen was inconclusive for the cause of necrosis. Five days post abdominal closure, the patient had increasing abdominal distension with an associated rise in inflammatory markers. A repeat CT scan demonstrated no significant abnormality and this was managed as a paralytic ileus. The patient subsequently recovered well in the ward with the reintroduction of diet and was discharged on day 12. He had no further complications and completed outpatient cardiac screening to exclude a thromboembolic cause for the small bowel ischemia, with echocardiography demonstrating no evidence of thrombus or regional wall abnormality.

## Discussion

Death due to Campylobacter enterocolitis is rare, with mortality rates estimated at 0.024% [7]. Campylobacter is a common foodborne pathogen, similar to Escherichia coli (E. coli), Salmonella, Yersinia, Shigella, and enteric viruses. Complications of bowel ischemia or perforation are more common from enterohaemorrhagic E. coli [8,9] and Salmonella typhi [10]. Previously reported gastrointestinal complications of Campylobacter enterocolitis include toxic megacolon [6], sepsis, and bowel obstruction [11]. Other non-gastrointestinal-related complications reported include myocarditis, pericarditis, Guillian-Barré syndrome, and reactive arthritis [12].

Campylobacter jejuni and Campylobacter coli are most commonly associated with enteric infections. Unfortunately, further information on the Campylobacter subtype was not available on our patients as only enteric PCR on stool specimens was performed. Immunohistochemistry from anatomical specimens was also not available.

The most commonly described symptom of Campylobacter enterocolitis is a diarrheal illness in 99% of cases [11], along with abdominal pain, fevers, malaise, myalgia, nausea, and vomiting. A large proportion of patients may also have associated lower gastrointestinal bleeding [11]. In both cases presented here, patients demonstrated similar symptoms of gradually worsening abdominal pain and diarrhea despite antimicrobial treatment. Interestingly, CT imaging in our patients demonstrated pneumatosis of the small bowel with portal venous gas, which contributed to the clinical decision for surgical intervention.

PI secondary to Campylobacter infections have been presented by Verma [5], and Magaz Martínez et al. [7]. In the former study, as mentioned above, the patient experienced a positive outcome with conservative management. The patient in the latter study died from multiple organ failure and metabolic acidosis due to macrolide-sensitive, quinolone-resistant Campylobacter jejuni. Previous studies have shown that findings of PI with portal venous gas are associated with a 70% chance of bowel ischemia. When accompanied by sepsis and elevated lactate levels, patients with PI have a higher mortality risk [13]. This was consistent with our cases, as our first patient, unfortunately, passed away. However, we note that there were other factors such as age and pre-existing medical conditions that might have contributed to the poor outcome.

## Conclusions

We presented two cases of Campylobacter enterocolitis with CT features of PI and portal venous gas and subsequent ischemic bowel on laparotomy. Consistent with the literature, the presence of PI and portal venous gas on CT was associated with bowel necrosis. The presence of sepsis and higher lactate also likely contributed to a higher chance of mortality in the presence of PI.

Our case series contributes to the current literature regarding potential rare complications of the usually benign and self-limiting disease of Campylobacter enterocolitis. Further studies are required to devise methods to improve the management of infectious colitis and identify patients who require early surgical intervention to reduce morbidity and mortality.
